# The Effect of Diabetes Self-Management Education on Body Weight, Glycemic Control, and Other Metabolic Markers in Patients with Type 2 Diabetes Mellitus

**DOI:** 10.1155/2014/789761

**Published:** 2014-07-17

**Authors:** Chuang Yuan, Christopher W. K. Lai, Lawrence W. C. Chan, Meyrick Chow, Helen K. W. Law, Michael Ying

**Affiliations:** ^1^Department of Health Technology and Informatics, The Hong Kong Polytechnic University, Hung Hom, Kowloon, Hong Kong; ^2^School of Nursing, The Hong Kong Polytechnic University, Hung Hom, Kowloon, Hong Kong

## Abstract

*Aims.* To comprehensively evaluate the effect of a short-term diabetes self-management education (DSME) on metabolic markers and atherosclerotic parameters in patients with type 2 diabetes. *Methods.* 76 patients with type 2 diabetes were recruited in this study. They were divided into the intervention group (*n* = 36) and control group (*n* = 40). The patients in the intervention group received a 3-month intervention, including an 8-week education on self-management of diabetes mellitus and subsequent 4 weeks of practice of the self-management guidelines. The patients in the control group received standard advice on medical nutrition therapy. Metabolic markers, carotid intima-media thickness (CIMT), and carotid arterial stiffness (CAS) of the patients in both groups were assessed before and after the 3-month intervention. *Results.* There was a significant reduction in hemoglobin A1c (HbA1c, −0.2 ± 0.56% versus 0.08 ± 0.741%; *P* < 0.05) and body weight (−1.19 ± 1.39 kg versus −0.61 ± 2.04 kg; *P* < 0.05) in the intervention group as compared to the control group. However, no significant improvements were found in other metabolic markers, CIMT and CAS (*P* > 0.05). *Conclusions.* DSME can improve HbA1c and body weight in patients with type 2 diabetes.

## 1. Introduction

Diabetes mellitus, commonly type 2 diabetes mellitus, is an increasing health problem worldwide. It has been estimated that there will be 552 million patients with diabetes and 300 million people with impaired glucose tolerance in 2030 [1]. Diabetes mellitus is associated with various atherosclerotic complications, including cerebrovascular and cardiovascular diseases, causing significant morbidity and mortality.

Monitoring of metabolic markers, such as blood pressure, body weight, lipid profile, blood glucose, and HbA1c, is essential in the clinical management of patients with diabetes, because hypertension, obesity, and dyslipidemia are well-known risk factors of atherosclerosis and are common in diabetic patients [2]. Monitoring of these risk factors also helps in the evaluation of treatment response of the patients. In addition, carotid intima-media thickness (CIMT) and carotid arterial stiffness (CAS) are atherosclerotic parameters which are usually considered as the predictors of cardiovascular and cerebrovascular events [3, 4]. Patients with diabetes tend to have increased CIMT and CAS [5, 6]. Therefore, assessing these atherosclerotic parameters is important for patients with diabetes to evaluate the risk of cardiovascular and cerebrovascular events as well as treatment response.

As a long-term disease, diabetes mellitus needs lifetime care and management. However, 50–80% of patients with diabetes did not have enough skills and knowledge for self-care of the disease [7]. Therefore, diabetes self-management education (DSME) plays an important role in the clinical management of diabetes. Previous studies have shown that DSME improves homeostasis of metabolism of the patients, and healthy lifestyles prevent the development of atherosclerosis in patients with type 2 diabetes [8, 9]. However, there is limited information in the literature about the effects of DSME on both metabolic markers and atherosclerotic parameters. Therefore, this study was undertaken to comprehensively investigate the effect of DSME on metabolic markers and atherosclerotic parameters in patients with type 2 diabetes.

## 2. Methods

### 2.1. Subjects

In the present study, a total of 88 patients with type 2 diabetes were recruited from a local Chinese non-profit-making organisation for diabetics (Angel of Diabetic, Hong Kong). The patients were randomly assigned into the intervention group (*n* = 44) or the control group (*n* = 44). Blocked randomization was used in the present study, and allocation sequence was concealed from researchers and patients. The inclusion criteria included Chinese and adult (>18 years old) having type 2 diabetes for more than one year. Criteria for exclusion were attendance of previous diabetes self-care courses, radiotherapy of the neck, carotid endarterectomy, and carotid stenting. Among the 88 patients, 12 of them (8 in the intervention group and 4 in the control group) did not complete the DSME programme or did not attend the follow-up examination and thus were excluded from the study. Therefore, finally 76 patients with type 2 diabetes were included in the study (intervention group, *n* = 36; control group, *n* = 40).

This study was approved by the Human Subject Ethics Subcommittee of The Hong Kong Polytechnic University. Patients were informed with the details of the study and written consent was obtained from the patients before they participated in the study.

### 2.2. Intervention

The patients in the intervention group participated in a DSME programme in which the patients needed to attend a 2-hour lesson weekly for eight weeks and to follow the self-management guidelines of the education programme in the daily activities within the study period. The patients in the control group did not attend any lessons of the DSME. However, they still received standard advice on medical nutrition therapy. For the DSME programme, all the lessons were conducted by a certificated nutritionist and were focused on the skills and knowledge for healthy eating, being active, monitoring, taking medication, problem solving, reducing risks, and healthy coping (Table 1) [10].

### 2.3. Blood Test

Blood test was conducted twice for each patient. For each blood test, fasting venous blood sample was obtained from the patient by venipuncture to evaluate the whole blood level of metabolic markers: total triglyceride, total cholesterol, high density lipoprotein (HDL), low density lipoprotein (LDL), blood glucose, and hemoglobin A1c (HbA1c). All the metabolic marker analyses were conducted using an automated clinical chemistry analyzer (Dimension Xpand Plus, Siemens Healthcare, Germany), and the level of the metabolic markers were assayed by the corresponding reagent cartridges (Siemens Healthcare, Germany).

### 2.4. Blood Pressure Measurement and Ultrasound Examination

Each patient had two ultrasound examinations of the carotid artery. All ultrasound examinations were performed using the Esaote MyLab Twice ultrasound unit in conjunction with a 4–13 MHz linear transducer (Esaote, Genoa, Italy). Before the ultrasound examination, blood pressure of the patient was measured by a sphygmomanometer (Tensoval, Hartmann, Germany) at the left upper arm in sitting position after the patient had at least 10 minutes of rest. The systolic and diastolic pressures were then inputted into the ultrasound unit for the assessment of CAS.

**Figure 1:**
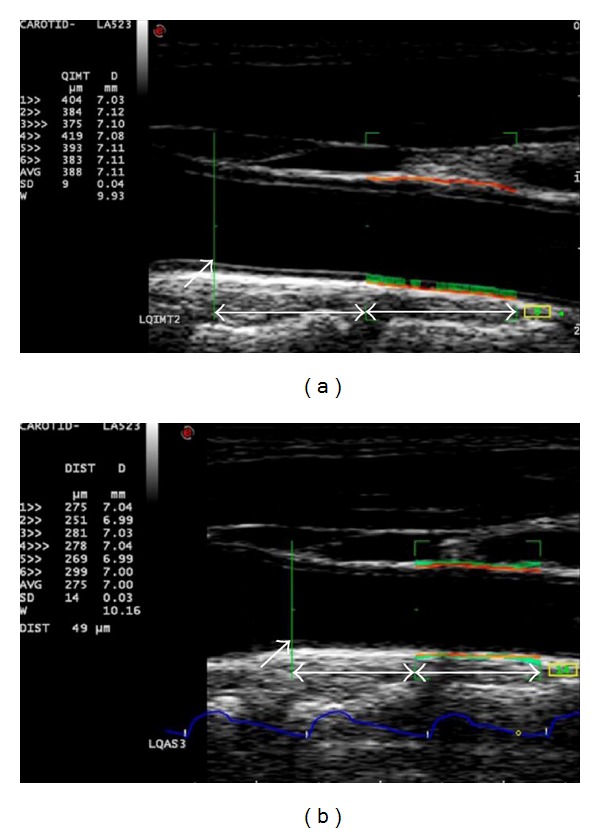
The assessment of intima-media thickness and arterial stiffness in the carotid artery. (a) Radiofrequency-based quality intima-media thickness. (b) Radiofrequency-based quality arterial stiffness. The arrows indicate the inferior end of the carotid bulb and the double-arrow lines show a distance of 1 cm.

The CIMT was evaluated with longitudinal scans of the CCA. CIMT was measured on the far wall over a 10 mm segment of the CCA from a point 10 mm proximal to the inferior end of the carotid bifurcation (Figure 1(a)). During the longitudinal scanning of the CCA, the transducer was slightly angled medially or laterally and rotated in clockwise or anticlockwise direction until a scan plane, which clearly demonstrated the carotid intima and media layers, was obtained. CIMT was measured using an automated quantification programme of the ultrasound unit, radiofrequency-based quality intima-media thickness (RF-QIMT) (Esaote, Genoa, Italy), which automatically identified the lumen-intima interface and media-adventitia interface of the CCA for measuring the CIMT (Figure 1(a)).

Similar to the measurement of CIMT, CAS was measured at the same segment of the CCA in the longitudinal scans. Scanning was performed carefully until a scan plane, which clearly shows the near and far walls of the CCA and demonstrated the CCA with maximum and uniform lumen diameter along the artery, was obtained. CAS was measured using an automated quantification programme of the ultrasound unit, radiofrequency-based quality arterial stiffness (RF-QAS) (Esaote, Genoa, Italy) (Figure 1(b)). The RF-QAS uses the echo-tracking technique, which tracks the movement of the near and far walls of the pulsating CCA during the scanning and measures the changes of the artery diameter during the pulsation. With the systolic and diastolic blood pressure values of the patients inputted to the ultrasound unit, the distensibility (compliance) and stiffness of the artery were automatically evaluated by the ultrasound system. In the evaluation of carotid arterial stiffness, five stiffness parameters were investigated: (1) distensibility coefficient (DC); (2) compliance coefficient (CC); (3) index *α*; (4) index *β*; and (5) pulse wave velocity (PWV). The higher *α*, *β* or PWV and the lower DC and CC are, the stiffer the carotid artery is. The stiffness parameters were calculated by the ultrasound system after the tracking of the arterial walls during the scanning.

Each carotid artery was scanned and measured three times, and the mean value of CIMT and CAS measurements were used for the data analyses. All the ultrasound examinations were performed by the same operator, and the operator was blinded to the grouping of the patients.

For the patients in the intervention group, blood tests and ultrasound examinations were performed before the commencement of the DSME programme as the baseline and one month after the completion of the DSME programme. For the patients in the control group, they had two blood tests and two ultrasound examinations with a time interval of three months.

### 2.5. Statistical Analysis

The continuous data was expressed as mean ± standard deviation (SD). Shapiro-Wilk test was used for checking the normality of distribution. If the data was normally distributed, *t*-test was used. Otherwise, nonparametric tests were applied. Demographic data and baseline characteristics between the intervention and control groups were compared using *χ* ^2^ test, *t*-test, or Mann-Whitney *U* test. Paired *t*-test or Wilcoxon Signed Ranks test were utilized to compare the measurements between the baseline and follow-up examinations of the patients. The differences between the intervention and control groups in the changes from the baseline to the follow-up examinations were determined using *t*-test or Mann-Whitney *U* test. All the data analyses were performed using Statistical Product and Service Solutions (SPSS) version 20 (IBM, Armonk, New York, United States). A *P* value < 0.05 was considered to be significant.

## 3. Results

### 3.1. Demographic Data

There was no significant difference in age, gender, duration of type 2 diabetes, metabolic markers, CIMT, and CAS between the intervention and control groups at the baseline assessment (*P* > 0.05, Table 2).

### 3.2. Metabolic Markers

For the patients in the intervention group, there was a significant decrease in the HbA1c level and body weight in the follow-up examination when compared with the baseline examination (*P* < 0.05, Table 2). However, similar observation was not found in the control group (*P* > 0.05, Table 2). The change of HbA1c and body weight after 3 month was significantly greater in the intervention group than in the control group (*P* < 0.05, Table 3).

For both intervention and control groups, there was a significant decrease in the total cholesterol, LDL, and BMI in the follow-up examination (*P* < 0.05, Table 2), but the improvements in the total cholesterol, LDL, and BMI after 3 months were not significant between the intervention and control groups (*P* > 0.05, Table 3). The blood glucose, triglyceride, HDL, SBP, and DBP levels were also not significantly different between the two study groups (*P* > 0.05, Table 3).

### 3.3. Atherosclerotic Parameters

In the intervention group, CIMT of the patients was significantly decreased in the follow-up examination (*P* < 0.05, Table 2). In contrast, there was no significant change in CIMT of the patients in the control group between the baseline and follow-up examinations (*P* > 0.05, Table 2). However, the difference in the change of CIMT after 3 months was not significant between the two groups (−23.3 ± 68.1 *μ*m versus −6.3 ± 71.4 *μ*m; *P* > 0.05; Table 3).

In both the intervention and control groups, there was no significant difference in CAS parameters (DC, CC, *α*, *β*, and PWV) between the baseline and follow-up examinations (*P* > 0.05, Table 2). The changes in these parameters after 3 months were also not significantly different between the two groups (Table 3, *P* > 0.05).

## 4. Discussion

The present study was a randomized and controlled clinical study which comprehensively evaluated the potential ameliorative effect of diabetes self-management education on metabolic markers and atherosclerotic parameters in patients with type 2 diabetes. In the present study, the self-management education given to the patients was of low intensity, which contained eight 2-hour sessions and encouraged the patients to follow guidelines instead of setting goals (e.g., weight loss) that patients should achieve. In spite of the low intensity, this education enabled the patients to systematically receive the information of self-management of type 2 diabetes mellitus. Our result showed that the patients in the intervention group had significant reduction of HbA1c level and body weight after receiving the education as compared to the control group, indicating that the education had positive effects for improving the health status of patients with type 2 diabetes.

The primary outcome of the present study is improved glycemic (in terms of reduced HbA1c) and body weight control of patients after receiving the DSME. HbA1c is an important indicator showing the severity of diabetes mellitus. Stratton et al. reported that each 1% reduction of the HbA1c level was related to a 37% reduction of microvascular complications, a 21% reduction of diabetes-related death and a 14% reduction of myocardial infarction [11]. Any reduction in the HbA1c level decreases the risk of diabetes-related complications [11]. In the present study, the HbA1c level in the intervention group was significantly decreased with a mean reduction of 0.2% after receiving the self-management education. In contrast, the HbA1c level in the control group did not show significant variation. The reduction of HbA1c after 3 month was significantly greater in the intervention group than in the control group. Thus, the results of the present study showed that DSME improved HbA1c control in patients with type 2 diabetes. The finding was consistent with previous study in which the HbA1c level of the diabetic patients was significantly decreased after receiving DSME [12].

Overweight is a common complication of DM and is associated with the development of atherosclerosis. It has been reported that ≥2% of weight loss in diabetic patients could mediate significant improvement of cardiovascular risk factors [13]. The DSME programme in the present study resulted in a significant weight loss in the intervention group (*P* < 0.05, Table 2) but not in the control group (*P* > 0.05, Table 2). The change of body weight after 3 months was significantly larger in the intervention group when compared with the control group (−1.89 ± 2.23% versus −0.77 ± 2.68%; *P* < 0.05). 44.4% of patients (16 in 36) in the intervention group lost ≥2% of initial weight, whereas only 22.5% (9 in 40) of patients in the control group achieved the improvement (*P* < 0.05). For the patients who had lost ≥2% of weight (*n* = 25), they had a significant improvement of HbA1c level when compared with the patients who had not lost ≥2% of weight (*n* = 51, −0.3 ± 0.93% versus 0.07 ± 0.44%; *P* < 0.05). These results suggested that the DSME programme in the present study resulted in significant weight loss which led to significant reduction of HbA1c level.

In contrast, DSME in the present study did not improve other metabolic markers. In both the intervention and control groups, there was significant decrease in the total cholesterol and LDL of the patients in the follow-up assessment. However, the changes in the total cholesterol and LDL during the study period were not significantly different between the intervention and control groups (*P* > 0.05, Table 3). Previous studies have reported that lipid profiles increased in colder seasons but decreased in warmer seasons [14]. The present study was conducted from March to June when the temperature was rising during the study period. Thus, we speculated that the variation of these parameters may be related to their seasonal changes rather than the effect of DSME. In addition, the changes in the triglyceride, HDL, plasma glucose, SBP, and DBP levels were also not significantly different between the two study groups (*P* > 0.05, Table 3). The intervention of the present study was in low intensity with 8 DSME lessons and had a relatively short time interval (3 months). Therefore, the effect of this DSME was only demonstrated as the change in homeostasis of HbA1c and body weight but not in the homeostasis of some other metabolic markers.

There were also no significant improvements in CIMT and CAS in the intervention group when compared with the control group (*P* > 0.05, Table 3). The negative findings in CIMT and CAS may attribute to the low intensity and short term of the intervention in the present study. Kim et al. conducted an intervention with intensive lifestyle modification which significantly decreased the progression of CIMT in type 2 diabetics [8]. In that study, the patients with type 2 diabetes in the intervention group were asked to receive a 16-lesson training involving healthy diet, exercise, and behaviors on a one-on-one training basis and to achieve goals such as reducing body weight to a certain level (5% of weight loss in obese subjects), undertaking sufficient physical activity (at least 150 min/week of brisk walking) and decreasing energy intake during a 6-month period. The intensive lifestyle modification reduced 1% of HbA1c and 40 *μ*m of CIMT, which was greater than those in the present study. However, the one-on-one training basis is time-consuming and not cost-effective and may not be feasible for all patients. The DSME programme used in the present study, even though was in low intensity, benefited the diabetic patients by significantly decreasing their HbA1c and body weight, and it is less time-consuming and more cost effective which could be more suitable for and acceptable by the patients.

There were limitations in the present study. Firstly, the time interval of the baseline and follow-up assessments was relatively short. Therefore, possible changes of CIMT, CAS, and some metabolic markers that are related to the DSME are not demonstrated. In addition, the long-term effect of the low intensity self-management education on diabetic patients was not fully evaluated in the present study. Moreover, the sample size of the present study was small with only 36 patients in the intervention group and 40 patients in the control group. Further investigations of the long-term effect of the DSME and with a larger sample size are suggested.

As a conclusion, DSME, even though in low intensity, significantly improved the glycemic and body weight control in patients with type 2 diabetes.

## Figures and Tables

**Table 1:** The content of the diabetes self-management education for the patients in the intervention group.

	Contents
Healthy eating	(i) Common misunderstanding of diet for self-management of type 2 diabetes mellitus
	(ii) The types of healthy and unhealthy foods for type 2 diabetics
	(iii) The benefits of health foods and the drawbacks of unhealthy foods for type 2 diabetics
	(iv) Recognition of healthy foods based on food package
	(v) Healthy cooking methods for type 2 diabetics
	(vi) Appropriate caloric intake for type 2 diabetics
	(vii) A brief method for counting calories of foods
	(viii) The best time to eat

Being active	(i) The importance of regular exercise for type 2 diabetics
	(ii) Common misunderstanding of exercise for self-management of type 2 diabetes mellitus
	(iii) Suitable types of exercise for type 2 diabetics
	(iv) Individualized plan for regular exercise
	(v) Self-check and control of body weight

Monitoring	(i) The importance of regular monitoring of blood glucose
	(ii) Methods of self-monitoring of blood glucose
	(iii) Self-management of blood glucose

Taking medication	(i) Pathology and medical treatments of type 2 diabetes mellitus
	(ii) The importance of taking diabetic medications
	(iii) Efficacies and side effects of different diabetic medications
	(iv) The appropriate time and frequency of taking diabetic medications

Problem solving	(i) Choosing healthy foods under various circumstances
	(ii) Doing appropriate exercises according to individual health status
	(iii) Methods to handle abnormal blood glucose

Reducing risks	(i) Common complications of type 2 diabetes mellitus
	(ii) Risk factors of diabetic complications
	(iii) The importance of stopping unhealthy behaviors (e.g., smoking) and maintaining healthy lifestyles

Healthy coping	(i) The importance of self-management of type 2 diabetes mellitus
	(ii) Designing an individualized plan for self-management of type 2 diabetes mellitus

**Table 2:** Comparison of ultrasound parameters of the carotid artery and metabolic markers between the baseline and follow-up assessments in the intervention and control groups.

	Intervention (*n* = 44)			Control (*n* = 44)		
	^ #^Baseline	Follow up	*P* value	^ #^Baseline	Follow up	*P* value
Age, years	58.9 ± 8.4	—	—	57.8 ± 8.2	—	—
Gender, female/male, *n*	22/14	—	—	28/12	—	—
Duration of diabetes, years	8.7 ± 6.9	—	—	7.3 ± 6.4	—	—
IMT, *μ*m	702.3 ± 127.7	678.9 ± 126.2	0.025∗	693.4 ± 127.3	687.1 ± 135.5	0.579
DC, 1/KPa	0.017 ± 0.009	0.016 ± 0.005	0.821	0.016 ± 0.006	0.017 ± 0.006	0.529
CC, mm^2^/KPa	0.731 ± 0.284	0.730 ± 0.266	0.660	0.753 ± 0.262	0.735 ± 0.279	0.525
*α*	6.081 ± 2.190	5.551 ± 1.784	0.122	5.551 ± 1.558	5.5597 ± 1.800	0.124
*β*	12.373 ± 4.418	11.280 ± 3.612	0.099	11.289 ± 3.148	11.400 ± 3.640	0.677
PWV, m/s	8.428 ± 1.618	8.078 ± 1.487	0.177	7.911 ± 1.177	8.028 ± 1.436	0.584
Blood glucose, mmol/L	7.689 ± 1.639	7.517 ± 1.602	0.238	7.968 ± 1.729	8.128 ± 1.771	0.427
Total cholesterol, mmol/L	4.664 ± 0.944	4.440 ± 0.961	0.034∗	4.865 ± 0.879	4.550 ± 0.714	<0.001∗
HDL, mmol/L	1.320 ± 0.297	1.343 ± 0.325	0.160	1.320 ± 0.337	1.281 ± 0.340	0.303
LDL, mmol/L	2.810 ± 0.768	2.590 ± 0.844	0.005∗	2.927 ± 0.748	2.631 ± 0.674	<0.001∗
Triglyceride, mmol/L	1.162 ± 0.644	1.112 ± 0.591	0.626	1.345 ± 0.841	1.389 ± 0.753	0.850
HbA1c, %	6.970 ± 0.915	6.772 ± 0.767	0.039∗	7.038 ± 1.042	7.118 ± 1.300	0.102
Weight, kg	60.25 ± 9.54	59.06 ± 9.03	<0.001∗	65.66 ± 13.38	65.05 ± 12.71	0.066
BMI, kg/m^2^	23.82 ± 4.57	23.25 ± 4.14	<0.001∗	25.42 ± 4.65	25.03 ± 4.35	0.019∗
SBP, mmHg	127.1 ± 17.7	125.9 ± 16.1	0.692	122.5 ± 14.8	124.8 ± 17.6	0.498
DBP, mmHg	75.3 ± 9.0	75.2 ± 7.3	0.916	73.0 ± 10.1	75.1 ± 10.2	0.221

^#^No significant difference (*P* > 0.05) was found in age, gender, duration of type 2 diabetes, metabolic markers, CIMT, and CAS between the intervention and control groups in the baseline assessment.

*P value indicates significant difference between baseline and follow-up examination in either intervention group or control group. IMT: intima-media thickness; DC: distensibility coefficient; CC: compliance coefficient; PWV: pulse wave velocity; HDL: high density lipoprotein; LDL: low density lipoprotein; HbA1c: hemoglobin A1c; BMI: body mass index; SBP: systolic blood pressure; and DBP: diastolic blood pressure.

**Table 3:** Change of characteristics of subjects from baseline to follow-up assessments in the intervention and control groups.

Characteristics	Intervention *n* = 36	Control *n* = 40	*P* value
ΔIMT, *μ*m	−23.3 ± 68.1	−6.3 ± 71.4	0.298
ΔDC, 1/KPa	−0.002 ± 0.01	0.001 ± 0.008	0.317
ΔCC, mm^2^/KPa	0.018 ± 0.200	−0.012 ± 0.236	0.487
Δ*α*	−0.531 ± 2.018	0.046 ± 1.637	0.185
Δ*β*	−1.093 ± 4.009	0.118 ± 3.334	0.193
ΔPWV, m/s	−0.351 ± 1.530	0.117 ± 1.339	0.159
ΔBlood glucose, mmol/L	−0.172 ± 1.400	0.160 ± 0.956	0.149
ΔTotal cholesterol, mmol/L	−0.219 ± 0.546	−0.315 ± 0.527	0.351
ΔHDL, mmol/L	0.024 ± 0.161	−0.039 ± 0.173	0.596
ΔLDL, mmol/L	−0.220 ± 0.446	−0.296 ± 0.489	0.106
ΔTriglyceride, mmol/L	−0.050 ± 0.425	0.044 ± 0.583	0.643
ΔHbA1c, %	−0.200 ± 0.560	0.080 ± 0.741	0.004∗
ΔWeight, kg	−1.19 ± 1.39	−0.61 ± 2.04	0.036∗
ΔBMI, kg/m^2^	−0.57 ± 1.00	−0.39 ± 1.00	0.105
ΔSBP, mmHg	−1.36 ± 16.70	2.34 ± 15.24	0.388
ΔDBP, mmHg	−0.05 ± 9.54	2.25 ± 9.74	0.223

*P value indicates significant difference in changes of variables between the intervention group and control groups. IMT: intima-media thickness; DC: distensibility coefficient; CC: compliance coefficient; PWV: pulse wave velocity; HDL: high density lipoprotein; LDL: low density lipoprotein; HbA1c: hemoglobin A1c; BMI: body mass index; SBP: systolic blood pressure; and DBP: diastolic blood pressure.
